# Fibroblast migration correlates with matrix softness. A study in knob-hole engineered fibrin

**DOI:** 10.1063/1.5022841

**Published:** 2018-07-17

**Authors:** Christopher Y. Leon-Valdivieso, Jennifer Wedgwood, Enrique Lallana, Roberto Donno, Iwan Roberts, Matilde Ghibaudi, Annalisa Tirella, Nicola Tirelli

**Affiliations:** 1 School of Materials, University of Manchester, Oxford Road, Manchester M13 9PT, United Kingdom; 2 Division of Pharmacy and Optometry, School of Health Science, University of Manchester, Oxford Road, Manchester M13 9PT, United Kingdom; 3 Laboratory of Polymers and Biomaterials, Fondazione Istituto Italiano di Tecnologia, via Morego 30, 16163 Genova, Italy; 4 Neurobiology of miRNA, Fondazione Istituto Italiano di Tecnologia, via Morego 30, 16163 Genova, Italy

## Abstract

The invasion of a matrix by migrating cells is a key step in its remodelling. At least in 2D migration models, cells tend to localize in stiffer areas (durotaxis). Here, we show that mechanical properties affect differently the 3D migration rate: non-proteolytic 3D cell migration is facilitated in softer matrices. In these gels, the modulus was varied by introducing defects in fibres, leaving largely intact the nanostructure. The matrices derive from fibrin via functionalization with a bioinert polymer [poly(ethylene glycol), PEG] through an affinity mechanism identical to that presiding to fibrin own self-assembly. Peptidic end groups on PEG were used to bind fibrinogen globular D regions [GPRP (glycine-proline-arginine-proline) for *a* holes, GHRP (glycine-histidine-arginine-proline) for *b* holes; K_d_ evaluated via isothermal titration calorimetry or fluorescence anisotropy]. In a dose-dependent manner, both PEGylated peptides decreased gel stiffness, but most other properties at a macroscopic [e.g., overall elastic character, strain hardening, and high (>0.5) Poisson ratio] or nano/micro level (fibre dimension and pore size) were largely unaffected, suggesting that the softening effect was due to the introduction of defects within fibres, rather than to differences in the network architecture. In these matrices, the key determinant of fibroblast migration was found to be the elastic modulus, rather than the identity or the dose of the PEGylated peptide; softer materials allowed a faster invasion, even if this meant a higher content of non-adhesive PEG. This does not conflict with fibroblast durotaxis (where stiffness controls accumulation but not necessarily the speed of migration) and indicates a way to fine tune the speed of cell colonization.

## I. INTRODUCTION

Fibrin is the provisional matrix *par excellence*, combining a rapid and responsive formation (during blood clotting) with tissue/cell adhesion and easy remodelling. It also provides morphological and chemical clues that control cell infiltration^1^ and guide the ensuing tissue remodelling processes.^2^ This has led to an extensive clinical and biomedical use, e.g., as a tissue sealant^3^ or as a matrix for tissue engineering.^4^ Possibly its best known drawback is its fast remodelling: rapid degradation is accompanied by macroscopic, cell-mediated contraction, ultimately leading to non-functional constructs.^5^ Protease inhibitors such as aminocaproic acid, aprotinin, or matrix metalloproteinase (MMP) inhibitors^6^ are typically used to ease these problems; this issue can also be tackled upstream, by controlling the rate of cell invasion, e.g., via engineering the matrix structure. Acting on clotting components allows a certain degree of control over architecture,^7,8^ since features such as fibre size or degree of branching are known to depend on pH, ionic strength, and on the concentration of fibrinogen, thrombin, Ca^2+^, and factor XIIIa.^9^ Other approaches have employed cross-linkers such as genipin^10^ or secondary scaffolds where fibrin is interpenetrated^11^ or present as a dispersed phase,^12^ but they often lack precise molecular control.

Here, we have followed two concepts: (a) affinity-based fibrin engineering. We have used knob-hole interactions (see below) to incorporate artificial elements in a site-specific and non-covalent fashion. (b) poly(ethylene glycol) (PEG) as a first example of artificial component: PEG is virtually devoid of any biological interactions, and in moderate amounts (up to five chains per fibrinogen), it only modestly affects fibrin clottability and enzymatic degradability;^13^ yet, its hydrophilicity and “stealth” character may affect cell migration.

**SCHEME 1.**
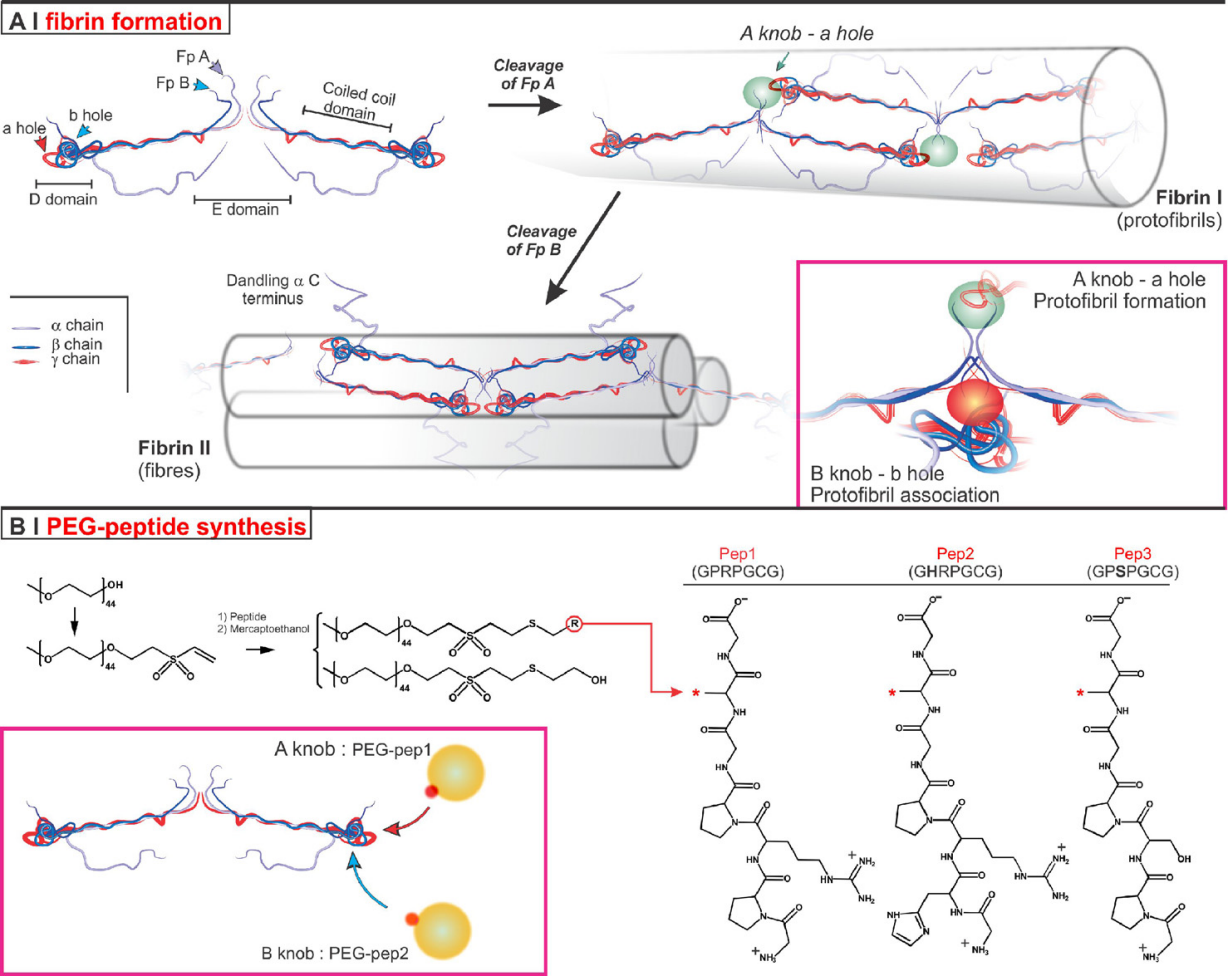
(a) Fibrinogen (top left) features a central globular part (E domain), consisting of the N-terminal regions of the three polypeptide chains; the E domain is linked through α-helical coiled-coil structures to two outer globular parts, referred to as D domains. The C-terminal regions of the β and γ chains are located in the D domains, whereas those of the Aα chains (in purple) fold back to bind sites in the E domain. During fibrin formation, thrombin transforms fibrinogen in two steps. The knob:hole interactions are at the basis both of fibrin polymerisation (A-knob:a-hole, highlighted as a green oval) and of the successive fibre formation (B-knob:b-hole later, highlighted as a red oval). Molecular mechanisms and hierarchical details are most extensively reviewed by Brown and Barker.^2^ (b) Preparation of PEG-peptide conjugates: OH-terminated PEG is transformed in PEG-VS via catalytic deprotonation with NaH and Michael-type addition of the resulting alcoholates onto an excess of DVS. Cysteine-bearing peptides, i.e., Pep1, Pep2, and Pep3, then react with PEG-VS; in this case, any unreacted VS groups are quenched by the successive use of mercaptoethanol to yield a non-biofunctional PEG derivative (PEG-ME). PEG-pep1 and PEG-pep2 can bind to fibrinogen a- and b-holes, respectively, whereas the lack of arginine prevents PEG-pep3 binding.

Knob-hole interactions are at the basis of fibrin own self-assembly. The fibrinogen-fibrin conversion operated by thrombin has two main steps^2,14,15^ [Scheme 1(a)]; in the first, the cleavage of fibrinopeptides A (Fp A) exposes N-terminal glycine-proline-arginine (GPR) amino acid sequences (*A-knobs*) and the resulting structure (referred to as fibrin I, desAA fibrin or α-fibrin^16^) assembles into protofibrils (binding of A-knobs to complementary *a-holes* in other fibrin molecules). Once grown to 600–800 nm, protofibrils aggregate laterally; the thrombin-mediated cleavage of fibrinopeptides B (Fp B) exposes glycine-histidine-arginine (GHR) amino acid sequences (*B-knobs*) at the N-termini of Bβ-polypeptide chains, allowing this second fibrin structure (fibrin II, desAABB fibrin or αβ-fibrin^16^) to bind *b-holes* in other fibrin molecules within the protofibril.^2,14,15^ The fibrillar network is then stabilized by further lateral aggregation (intermolecular interactions between αC-domains of different fibrin molecules) and Ca^2+^-dependent covalent cross-linking by factor XIIIa (a plasma transglutaminase).^2,15–17^

The use of knob-hole interactions has been pioneered by the group of Barker and originally applied to the incorporation of therapeutic proteins in fibrin.^18^ In general, knob sequences are introduced onto artificial components such as PEG, that then associate to fibrin(ogen) during its clotting. The knob-hole PEGylation is particularly interesting, since it can introduce controlled defects without additional possibilities of interactions, and therefore allows for a tuneable modulation only of fibrin mechanical and nanostructural details. This potential is demonstrated by the inhibition of fibrin clotting by a large excess of mono-GPRP PEG, likely by reducing both the formation and the lateral aggregation of protofibrils; PEG's molecular weight is critical: the best inhibition with 5 kDa PEG is a compromise between capacity to bind fibrin (worse for larger PEGs) and hindrance to aggregation (worse for the smaller 2 kDa PEG).^19^ It has also been shown that B-knob (alanine-glycine-histidine-arginine, AHRP)-bearing 5 kDa PEG (on 4 mg/ml fibrin)^20^ increased fibrin modulus, whereas with A-knobs (GPRP) decreased it, probably because the former decorated and thus stiffened fibres, and the second interrupted them;^21^ the cell permissiveness (the angiogenic potential) of these gels was not significantly affected by the presence of either A- or B-knob PEGs. Somehow unexpectedly, A-knob(GPRP sequence)-terminated bi- and tetrafunctional PEGs—in principle fibrin cross-linkers—decreased the elastic modulus,^22^ reportedly due to reduced lateral aggregation of fibrils; in our opinion, increased hydrophilicity may also play a role.

Here, we have tackled the quantification of the mechanical effects of 2 kDa PEG (low molecular weight chosen not to hinder protofibril formation) with both A- and B-knobs and addressed the overarching question whether and how these details may affect and possibly control cell migration, using adult human dermal fibroblasts (HDFa) as a model of invading and contractile cells. It is worth mentioning that we have used the most classical a-hole (GPRP) and b-hole (GHRP)-binding peptide sequences^15,23–25^ [Scheme 1(b)]; the mammalian-derived GHRP sequence has been shown to be less selective for *b* holes than the chicken-derived AHRP,^26^ but nevertheless we have preferred GHRP as a more physiologically relevant (for interactions with human fibrinogen) mechanism. Finally, Pep3 presents a similar structure lacking arginine and therefore incapable of binding to fibrinogen holes.

## II. RESULTS AND DISCUSSION

### A. Preparation of PEG-peptides

PEG was modified with a terminal vinyl sulfone (PEG-VS) to react with the cysteine residues through Michael-type addition; the reaction partners were selected for their reactivity (quantitative conversion typically within an hour), the absence of side products and the stability of the reaction product: sulfones, unlike, e.g., acrylates, are not hydrolysable, and there is little risk of retro-Michael reaction, as for maleimides. PEG-VS was prepared from 2 kDa PEG-OH and an excess of divinylsulfone (DVS) according to a literature procedure,^29^ which minimizes the possible side reactions of alcoholates by using a catalytic amount of NaH as a non-nucleophilic base (see also supplementary material, Fig. 5SI).

PEG-VS was then reacted with peptides featuring a cysteine flanked by glycines (low hindrance to thiols) at the C terminus, and N-terminal GPRP (*a* hole binding), GHRP (*b* hole binding), and glycine-proline-serine-proline (GPSP, non-binding) sequences.

Two points are noteworthy in this reaction: (a) all peptides were reduced prior to the reaction, using diluted sodium borohydride in 1 M NaOH,^30^ followed by the Michael-type addition at pH = 9. (b) we have avoided the presence of free peptides in the final material (possibly competing with the conjugates and difficult to separate from them), by using an excess of PEG-VS (PEG-VS/thiol molar ratio = 1:0.8) to ensure that all peptides are linked to PEG, and then quenched this excess via the addition of mercaptoethanol (ME): this produces an alcohol-terminated PEG (PEG-ME) with no significant affinity to fibrin(ogen), and therefore no foreseen effects on fibrin gelation. The conversion of vinyl sulfone groups was quantitative for all PEG-peptide products (Table I), with always more than 70 mol. % of the final material composed of the PEG-peptides (the rest being PEG-ME).

**TABLE I.** Characterization data for PEG conjugates.

		^1^H NMR			K_d_ (*μ*M)^e^	
	Yield (wt.%)^a^	Pept./ME (mol %)^b^	$\bar{M_{n}}$ (g/mol)^c^	MALDI-ToF (*m/z*)^d^	ITC	FA
PEG-VS	72	…	2089	[M+Na]^+^ 2126	…	…
				[M+K]^+^ 2110		
PEG-pep1	66	80/20	2851	[M]^+^ 2686	103 ± 5 **Pep1:** 44 ± 9	135 ± 8 **Pep1:** 40 ± 1
PEG-pep2	60	86/14	2891	[M]^+^ 2770	Not detected **Pep2:** 351 ± 10	1.8± 0.6 × 10^3^ **Pep2:** 335 ± 6
PEG-pep3	90	70/30	2662	[M+Na]^+^ 2727[M+H]^+^ 2705	Not measurable **Pep3:** not detected	Not measurable **Pep3:** not detected

^a^ Calculated as the weight of recovered material/theoretical weight of the reaction product.

^b^ Proton nuclear magnetic resonance (^1^H-NMR) spectra, chemical structures, and proton numbering are reported in supplementary material, Fig. 7SI. The ratio between peptide- and ME-terminated PEG is calculated from the ratio of the resonance of a PEG-ME methylene at 2.84 ppm (protons A, in α to the thioether and β to the terminal primary alcohol) and the PEG-pep proline main chain protons either at 4.54 (H19) and 4.47 (H11) ppm (for PEG-pep1), at 4.48 (H11) ppm (for PEG-pep2) or at 4.57–4.45 (H11, H17) ppm (for PEG-pep3); the vinyl sulfone peak has completely disappeared.

^c^ PEG-VS samples: $\bar{M_{n}}$ calculated using the number of repeating units calculated by averaging the ratio of the ^1^H NMR resonance of the PEG-VS terminal groups (methylene and ethylene protons at 3.25 ppm and 6.1/6.4 ppm) and those in the main chain at 3.5–3.8 ppm. PEG-peptide samples:$\;\bar{M_{n}}$ calculated from the ratio of the ^1^H NMR resonances of terminal groups [obtained averaging the PEG-ME methylene (protons A) or PEG-peptides proline protons (H11, H17, H19)], and those of PEG main chain.

^d^ Most intense peaks in MALDI-ToF (Matrix-Assisted Laser Desorption/Ionization-Time of Flight) spectra; all other peaks in the distribution are spaced by an additive 44 mass atomic units (see also supplementary material, Fig. 6SI).

^e^ K_d_ values obtained through fluorescence anisotropy (FA) and isothermal titration calorimetry (ITC) as described in the Methods section and in supplementary material, Sec. 1SI. The values obtained for non-conjugated peptides are also reported; note that Pep3 showing no binding, no competition assay is possible for PEG-pep3. *n *=* *3 independent samples for both techniques.

### B. Affinity of peptides to fibrinogen

**FIG. 1.**
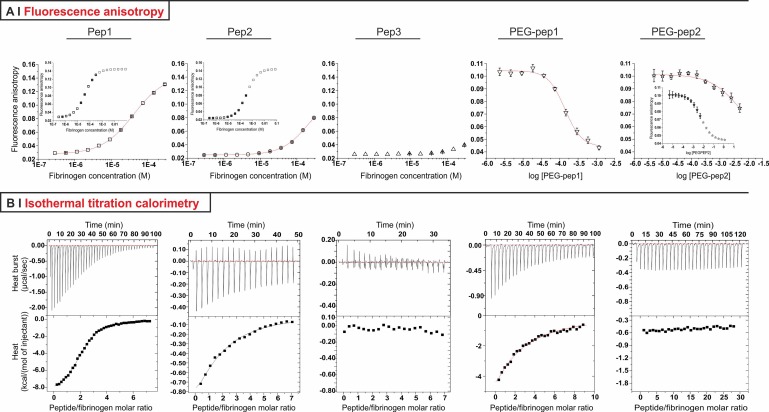
(a) FA curves for the binding of fluorescently labelled peptides 1, 2, and 3 to fibrinogen and for the competitive binding of PEG-pep1 and PEG-pep2 (displacement of 5IAF-pep1 and 5IAF-pep2 from fibrinogen, respectively). Insets present the results of fitting procedures with a Hill model for Pep1 and Pep2, and with a dose-response model for PEG-pep2. (b) ITC profiles for the binding of Pep1, 2 and 3, and PEG-pep1 and 2 to fibrinogen. Raw data (top) and integrated heats (bottom) are presented in all cases.

Two techniques were used to confirm that PEG-peptides retained the ability to bind to fibrinogen (Fig. 1 and Table I, right): Fluorescence Anisotropy (FA) and Isothermal Titration Calorimetry (ITC). In FA measurements [Fig. 1(a)], the dissociation constant (K_d_) was determined via saturation experiments for the non-conjugated peptides, and via competition experiments for the PEG-peptides. In the first case, saturation was achieved by adding increasing amounts of fibrinogen to a given amount of fluorescein-labelled peptides. In competition experiments, fibrinogen was pre-complexed with fluorescein-labelled peptides; these low molecular weight fluorophores were displaced upon addition of non-labelled conjugates, with a corresponding decrease in the polarization of the emitted light (smaller tumbling time). Note that, since saturation experiments showed that Pep3p had virtually no affinity [Fig. 1(a), centre], no competition experiment was possible, and it was assumed PEG-pep3 to have no measurable affinity for fibrinogen.

Both through FA and ITC, Pep1 presented roughly one order of magnitude higher affinity to fibrinogen than Pep2, with K_d_ values in the micromolar range; this is broadly in accordance with literature reports on similar peptides.^15,23,25^ In conjugates, the steric hindrance caused by PEGylation markedly decreased the affinity of Pep1 and 2 for fibrinogen, but did not change their relative ranking: although PEG-pep2 was analysable only through FA, its affinity for fibrinogen was still about one order of magnitude lower than that of PEG-Pep1; it is worth noting that this is a generic affinity, as neither FA not ITC allow to discriminated between a or b holes. Therefore, it could only be hypothesized PEG-pep2's mode of binding to be similar to that of Pep2, i.e., possibly intervening at a later stage and affecting more lateral aggregation than protofibril formation.

### C. Effect of PEG-peptides on fibrin gelation kinetics and mechanical properties

We have produced samples with variable fibrinogen concentration, but constant thrombin concentration, thus increasing the fibrinogen/thrombin ratio with fibrinogen concentration. Therefore, although the absolute rate of fibrinopeptide cleavage increased, it remained constant in relative terms: at any given time the same mol. % of fibrinopeptides is cleaved for all formulations, so any change in gelation kinetics is to ascribe to events of the self-assembly phase. We also would like to point out that at the highest fibrinogen concentrations (25 and 50 mg/ml) used, some of the effects presented here may be due to hindered thrombin diffusion due to the high viscosity of the solution rather than the thermodynamics of self-assembly.

First, we have used oscillatory rheology to study the gel point, the G′/G″ values at plateau and the behaviour of the resulting gels at high strains.

**FIG. 2.**
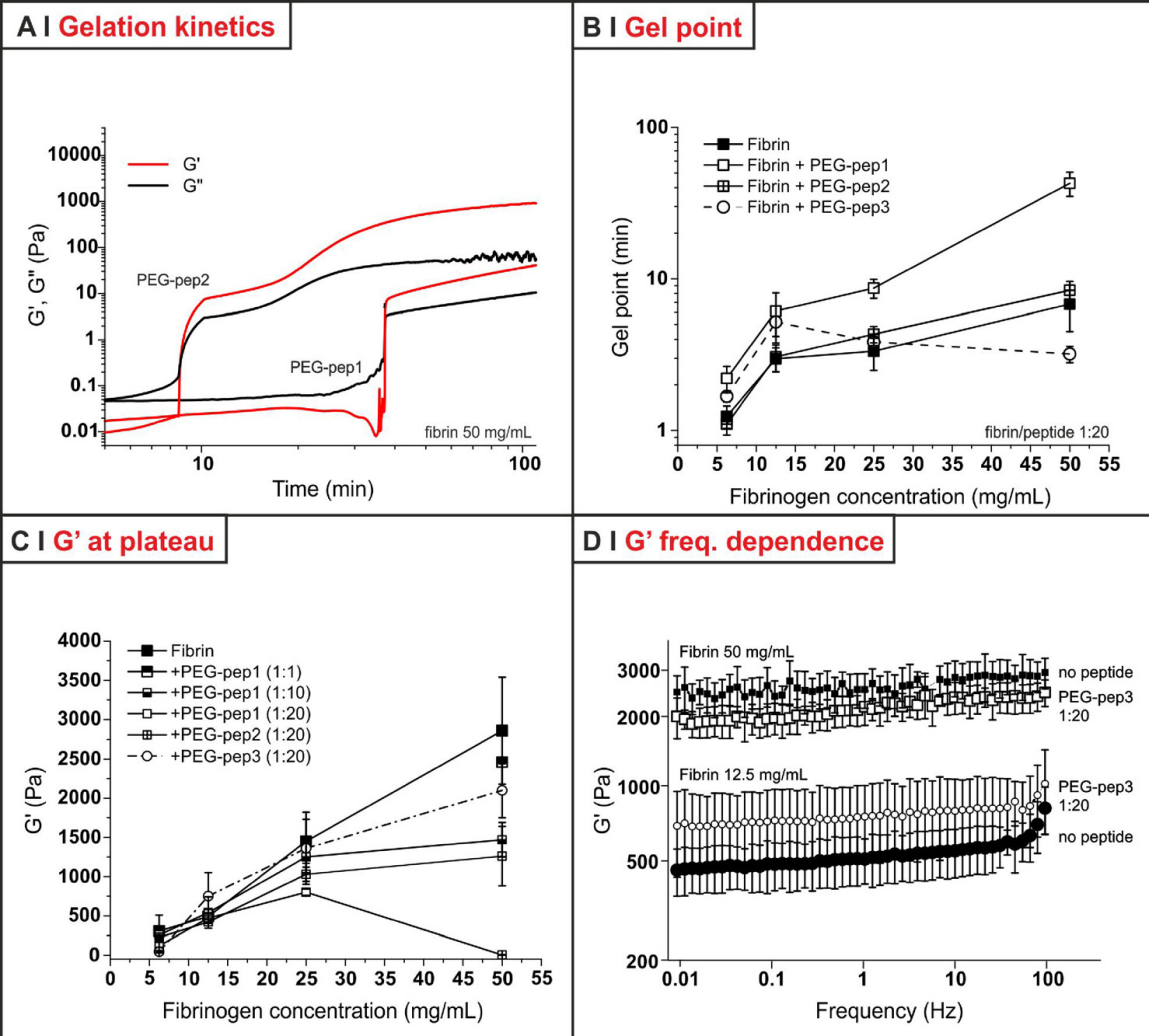
(a) Storage (G′) and loss (G″) modulus (stress = 5 Pa, frequency = 1 Hz) as a function of time for two PEG-peptides mixtures with fibrinogen (1:20 molar ratio to fibrinogen 50 mg/ml). (b) Gel point values (time at which G′ = G″; stress = 5 Pa, frequency = 1 Hz) for fibrin gels with and without PEG-peptides (fibrinogen/PEG-peptide molar ratio of 1:20, corresponding to a PEG peptide concentration of 1.0, 2.0, 4.0, and 7.9 mg/ml for fibrinogen 6.25 12.5, 25, and 50 mg/ml, respectively). Note that at a fibrinogen concentration of 6.25 mg/ml, all gel points are statistically indistinguishable. Data represent the mean ± standard deviation of independent samples (*n *=* *3). (c) Plateau (time > 100 min) values of storage shear modulus (G′) for fibrin gels with and without PEG-peptides (stress = 5 Pa, frequency = 1 Hz; fibrinogen/PEG-peptide molar ratio in the legend); the effect of the latter not only had no trend (at 12.5 mg/ml PEG-pep3 caused a small increase in the average value of G′, at 50 mg/ml a small decrease), but is also barely significant. (d) Plateau values of G′ as a function of frequency, showing that PEG-pep3 did not introduce any statistically significant effect both at an intermediate (12.5 mg/ml) and a high (50 mg/ml) concentration of fibrin.

All formulations, with or without PEG-peptides, showed well recognizable gel points [crossing of G′ and G″; Fig. 2(a)]. It is noteworthy that fibrin gelation does not depend only on the speed of chemical events, but also on the network morphology: for example, higher fibrinogen concentrations produce smaller but more dense and branched fibres;^31,32^ therefore, despite a larger number of interacting sites, concentrated samples gel slower.^8^

This dependence of gel time on fibrinogen concentration was not affected by the presence of PEG-pep1 or PEG-pep2 [Fig. 2(b)]. However, PEG-pep1 clearly slowed the process down; in a dose-dependent fashion, it also decreased the final G′ value of the gels, with the highest effect seen at a concentration of 50 mg/ml and a 1:20 fibrinogen/PEG-peptide molar ratio [Fig. 2(c)]. This effect is not surprising: GPRP alone is already known to significantly reduce fibrin clotting at a 1:20 fibrinogen/peptide molar ratio and to completely prevent it above 1:100.^24,25^ On the contrary, PEG-pep2 did not seem to affect the gelation time at any of the four fibrinogen concentrations tested; we ascribe this to the late onset rather to the low intensity of B-knob:b-hole interactions, as G′ of 25 and 50 mg/ml fibrin was indeed reduced by PEG-pep2 [Fig. 2(c)], with the effect of 1:20 molar ratio PEG-pep2 being similar to what obtained with 1:10 PEG-pep1. It is worth noting that at the highest fibrin concentration the non-binding PEG-pep3 had an accelerating effect [lowest gel point, Fig. 2(b)]; however, it also had negligible effects on G′, at any concentration and through an extended frequency range [Fig. 2(d)], which indicates that the fibrin network was not significantly affected by its presence. We thus ascribe the accelerated kinetics to partial PEG/fibrinogen immiscibility at high concentration, with a more rapid aggregation in the fibrinogen-rich phase.

All gels showed tan δ values < 0.1 (Table II) and a flat dependency of G′ on frequency, except 50 mg/ml fibrin/PEG-pep1 1:20, this behaviour is most typical of covalently cross-linked hydrogels;^33^ therefore, we do not feel to exclude the presence of transglutaminase (factor XIII) in our samples, as already noticed by other authors.^31^

**TABLE II.** Mechanical characterisation of the fibrin hydrogels produced in this study.

Fibrin conc. (mg/ml)	PEG-peptide (molar ratio)	Oscillatory measures^a^		Creep^d^			
		tan δ^b^	G′ (Pa)^c^	*G* (Pa)^e^	τ (s)^e^	${(J}_{m}{-J}_{0})/J_{0}$^f^	η (Pa s)
6.25	…	0.03 ± 0.02^¶^	305 ± 203	…	…	…	…
	PEG-pep1 (1:10)	0.02 ± 0.01^¶^	256 ± 90	…	…	…	…
	PEG-pep3 (1:20)	0.05 ± 0.04^¶^	46 ± 15	…	…	…	…
	PEG-pep2 (1:20)	0.02 ± 0.01^¶^	226 ± 52	…	…	…	…
	PEG-pep1 (1:20)	0.03 ± 0.01^¶^	119 ± 74	…	…	…	…
12.5	…	0.06 ± 0.01^¶^	490 ± 90	420 ± 90	1.8 ± 0.7	0.10 ± 0.02	…
	PEG-pep1 (1:10)	0.05 ± 0.01^¶^	540 ± 200	540 ± 100	0.9 ± 0.3	0.01 ± 0.05	…
	PEG-pep3 (1:20)	0.04 ± 0.01	750 ± 300	620 ± 120	1.6 ± 0.3	0.21 ± 0.15	…
	PEG-pep2 (1:20)	0.07 ± 0.01^¶^	420 ± 80	360 ± 40	0.4 ± 0.1	0.20 ± 0.04	(5 ± 2) × 10^4^
	PEG-pep1 (1:20)	0.07 ± 0.01^¶^	470 ± 60	390 ± 60	0.7 ± 0.1	0.23 ± 0.12	(5 ± 0.1) × 10^4^
25	…	0.05 ± 0.03	1450 ± 280	1240 ± 90	…	…	…
	PEG-pep1 (1:10)	0.05 ± 0.01	1250 ± 200	1050 ± 190	…	…	…
	PEG-pep3 (1:20)	0.05 ± 0.02^¶^	1360 ± 460	1080 ± 40	…	…	…
	PEG-pep2 (1:20)	0.07 ± 0.02^¶^	1030 ± 90	840 ± 80	1.9 ± 0.02	0.21 ± 0.14	…
	PEG-pep1 (1:20)	0.08 ± 0.03^¶^	800 ± 40	690 ± 50	0.7 ± 0.1	0.14 ± 0.02	(12 ± 1) × 10^4^
50	…	0.07 ± 0.04	2860 ± 680	2370 ± 290	…	…	…
	PEG-pep1 (1:1)	0.05 ± 0.02	2460 ± 20	2200 ± 270	…	…	…
	PEG-pep1 (1:10)	0.06 ± 0.02	1470 ± 220	1230 ± 50	…	…	…
	PEG-pep3 (1:20)	0.06 ± 0.02	2100 ± 350	1640 ± 110	…	…	…
	PEG-pep2 (1:20)	0.08 ± 0.03^¶^	1260 ± 380	1100 ± 340	2.1 ± 0.2	0.28 ± 0.03	…
	PEG-pep1 (1:20)	0.48 ± 0.17^¶^	5 ± 3	4 ± 1	0.4 ± 0.1	0.72 ± 0.27	260 ± 150

^a^ Stress: 5 Pa.

^b^ Calculated as G″/G′, averaging their values over the 0.01–100 Hz or 0.1–10 Hz frequency range; samples labelled with the ^¶^ symbol showed a minor drift of G′ with frequency (*n *=* *3).

^c^ Average value at a frequency of 1 Hz (*n *=* *3).

^d^ Stress: 5 Pa, duration: 10 min (*n *=* *3).

^e^ G=1/J was obtained from the modified Standard Linear Solid (SLS) model^34^ $J\left(t\right)=J_{0}+J_{1}+J_{2}=J_{0}+J_{1}\left(1-\;\text{exp}\;\left(-\frac{t}{\tau }\right)\right)+\left(\frac{t}{\eta }\right)$, which also provided τ and η.

^f^ $J_{0}$ represents the purely elastic compliance at the beginning of the creep phase and $J_{m}$ the maximum compliance reached at the end of the creep phase (compliance at 10 min). The parameter ${(J}_{m}{-J}_{0})/J_{0}$ provides an information all-in-all similar to tan δ (viscoelastic and viscous vs. elastic contributions); when it can be calculated, it appears to be more sensitive than tan δ.

**FIG. 3.**
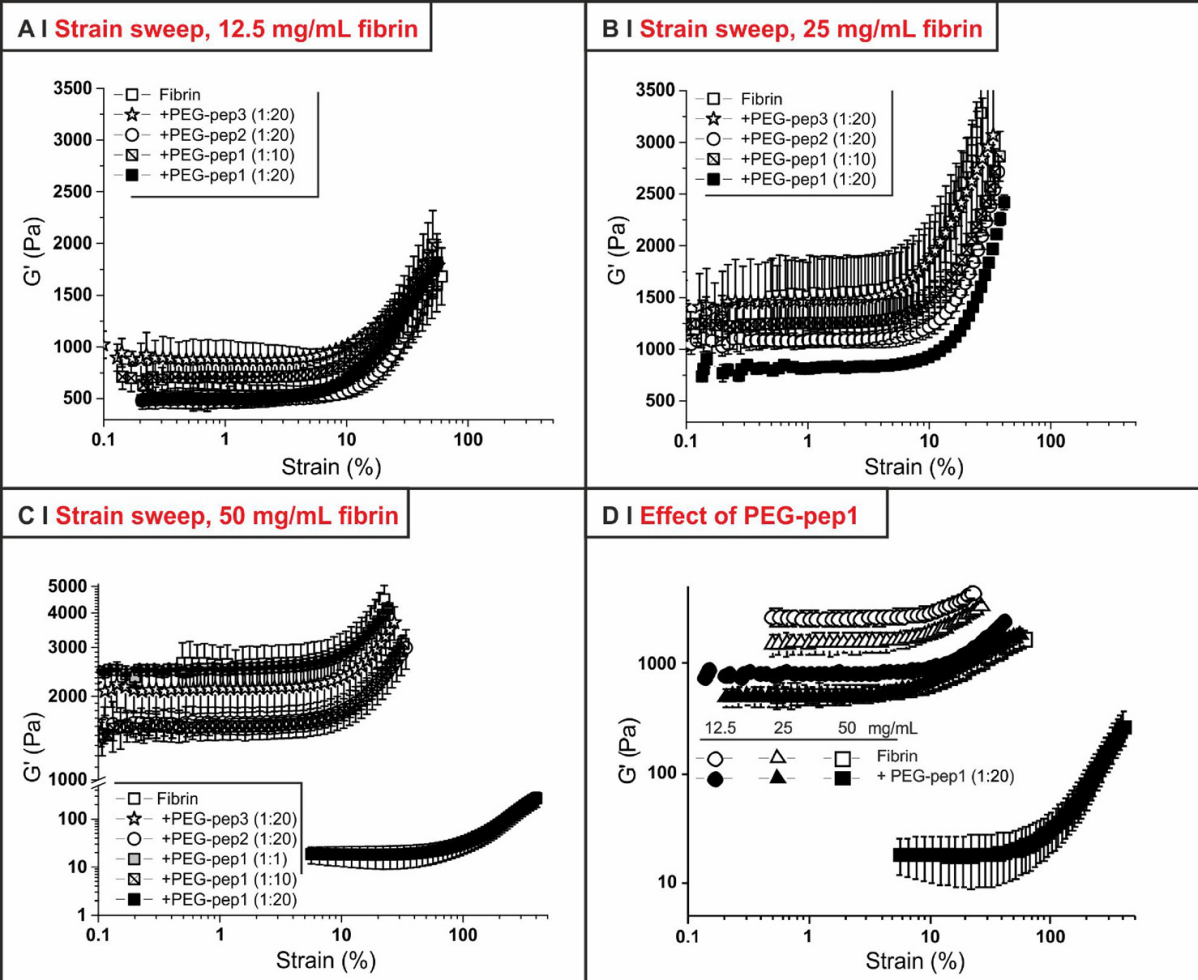
Stress amplitude tests (frequency = 1 Hz; shear stress = 1–1000 Pa; T = 37 °C) of hydrogels with different fibrinogen concentrations [12.5 (a), 25 (b), or 50 (c) mg/ml)]. Panel (d) focuses on the effect of PEG-pep1 at a 1:20 fibrinogen/PEG-peptide molar ratio. (*n *=* *3).

Fibrin gels are known to exhibit a distinct hardening at strain values above ∼10%,^35^ which is ascribed to intermolecular interactions of the αC region.^36^ Any alteration to this profile would suggest a different network organization, e.g., the more or less pronounced presence of fibre endings, changes in fibre diameter, or irregularities in the internal fibre packing. However, although decreasing G′ values as previously described, all but one PEG-peptide formulation did not alter the overall strain-hardening behaviour [Figs. 3(a)–3(c)]; the exception is the 50 mg/ml fibrin/PEG-pep1 (1:20) sample, whose large decrease in G′ caused the material to harden only at significantly higher strain [around 100%, Figs. 3(c) and 3(d)]. It would therefore appear that generally the network morphology and the nature of the inter-fibrillar interactions are substantially unchanged by the presence of PEG-pep2, or by that of the non-binding PEG-pep3, whereas PEG-pep1 may alter it, above all at high fibrin concentrations [Fig. 3(d)].

**FIG. 4.**
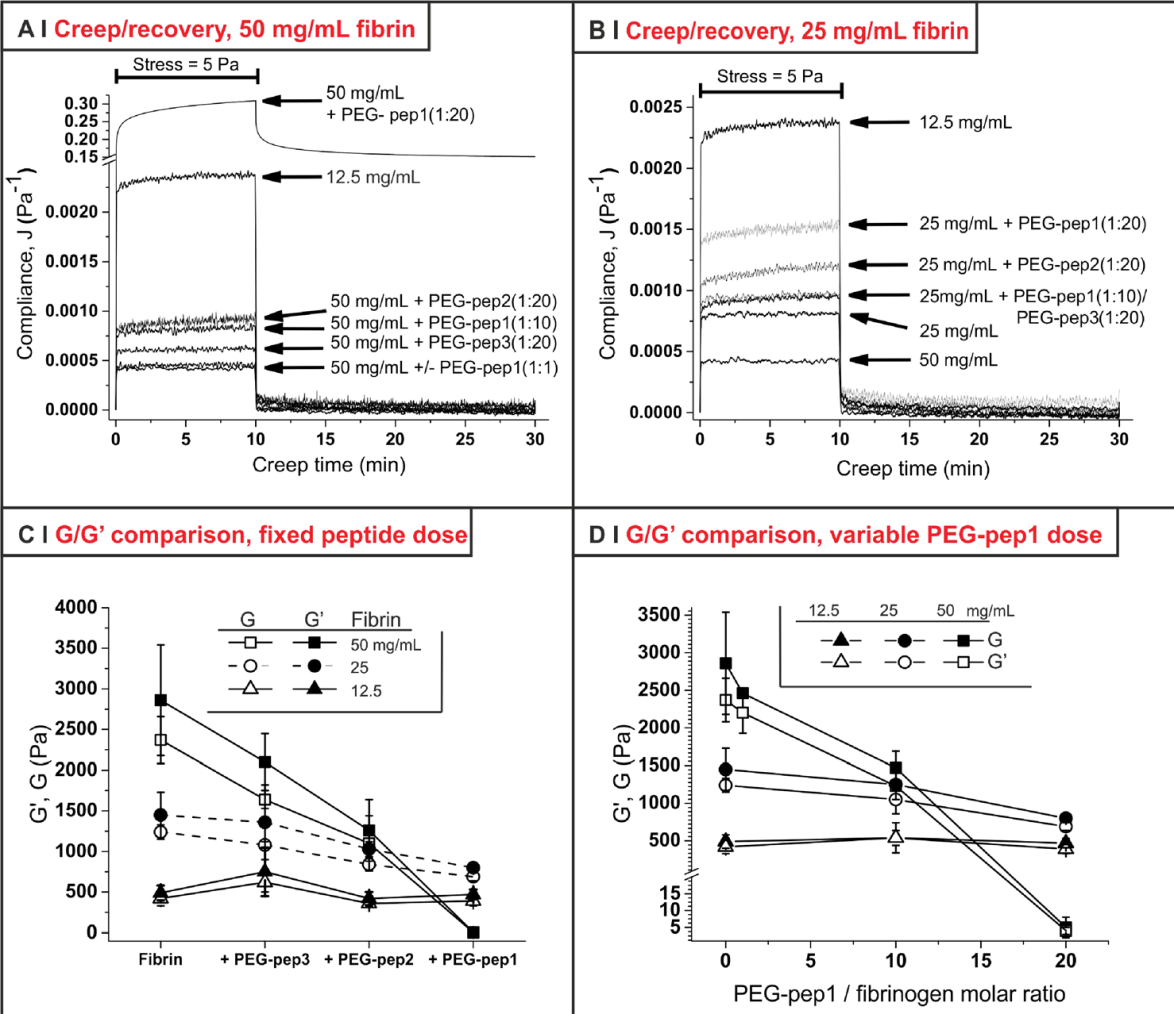
(a) Creep and recovery curves (steady shear stress = 5 Pa; creep time = 10 min; recovery time 20 min; T = 37 °C) for gels with a fibrin concentration of 50 mg/ml and various PEG-peptides; the creep and recovery behaviour of 12.5 mg/ml is reported to better appreciate the much increased compliance obtained with the highest amount of PEG-pep1. (b) As in (a), but for gels with a fibrin concentration of 25 mg/ml. (c) Dependence of creep shear modulus (G) and storage shear modulus (G′) on the nature of PEG-peptides for gels prepared with three different fibrin concentrations. (d) The dependence of G and G′ on the concentration of PEG-pep1 was dramatically different for the three fibrin concentrations, with almost negligible effects for 12.5 mg/ml and a very sharp dependency for 50 mg/ml. (*n *=* *3).

**FIG. 5.**
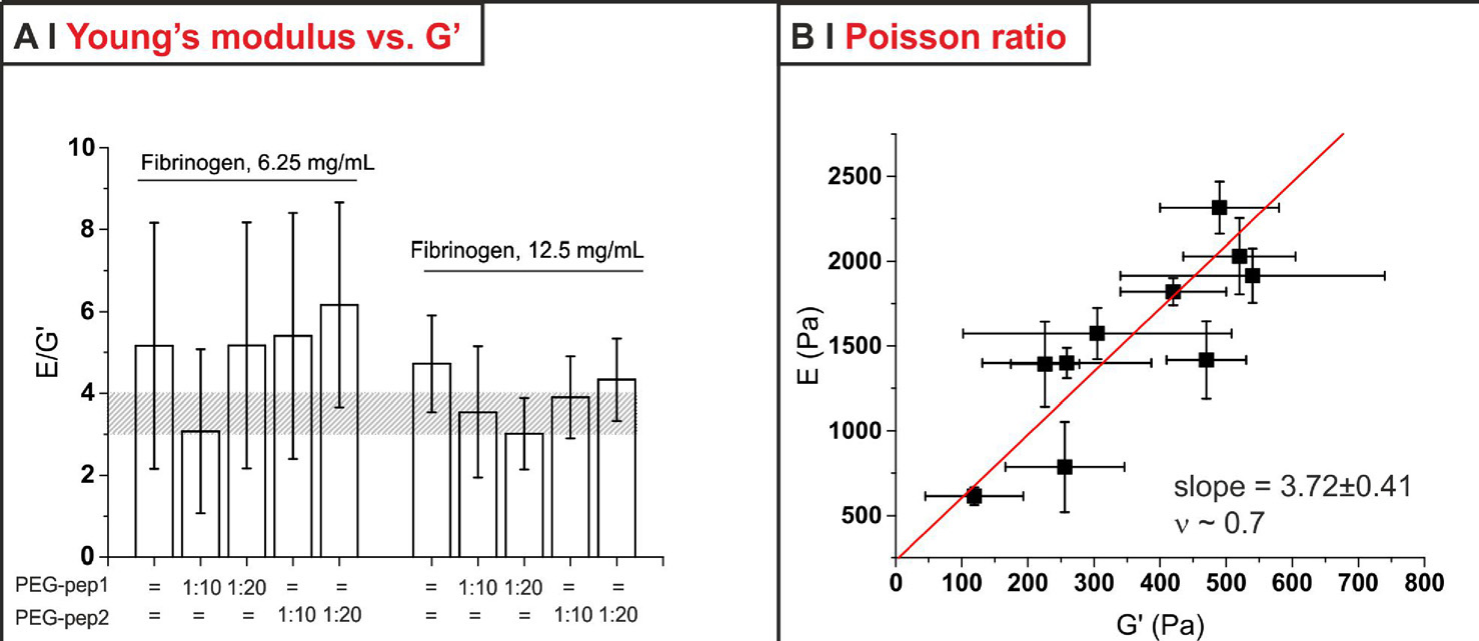
(a) The ratio between Young's modulus (E) from compression measurements and storage modulus (G′) from oscillatory shear experiments was substantially constant throughout all the 6.25 and 12.5 mg/ml fibrin formulations; please note that the large error bars stem predominantly from the relative error of the G′ measurements above all for the softest materials. It is, however, possible to see that in most cases E was 3.5–4 times larger (shaded area) than G′. (b) E and G′ data of the formulations in (a) can be correlated, in order to estimate a value for the Poisson ratio (ν) of these materials.

In order to confirm the results of oscillatory rheology with non-frequency-dependent measurements, the mechanical behaviour of the gels was also investigated in shear creep and recovery experiments [Table II, Figs. 4(a) and 4(b), see also supplementary material, Sec. 1.2SI and Fig. 1SI]. Note that gels made at 6.25 mg/ml of fibrinogen were not strong enough to bear creep at 5 Pa steady shear stress without breaking, and smaller stresses provided very noisy data; therefore, only higher fibrin concentrations were used in creep and recovery experiments, while the non-oscillatory analysis of softer gels was performed in compression tests (discussed later and shown in Fig. 5).

First, both fibrin and almost all fibrin/PEG-peptides formulations exhibited a clearly elastic behaviour with quantitative recovery, and a minor or negligible time-dependent (viscoelastic) component; the latter was recognizable at a low fibrin concentration and with a high PEG-peptide/fibrin molar ratio (see third last and second last columns in Table II). A viscous component could only be seen with 1:20 PEG-pep1 (all fibrin concentrations) and in fibrin 12.5 mg/ml with PEG-pep2 (still 1:20); however, in general, it accounted for a really minor non-recoverable deformation of the gels; as already seen, the behaviour 50 mg/ml PEG-pep1 1:20 was an exception, with a high non-recoverable component to compliance (low zero-shear viscosity, last column in Table II).

Second, we have fitted the creep results with a modified Standard Linear Solid model and obtained the shear modulus G of the materials (Table II); these data are substantially identical to those obtained with oscillatory measures at a frequency of 1 Hz [Figs. 4(c) and 4(d)]. For the softer formulations, whose creep and recovery analysis did not always provide satisfactory results, we have used stress-strain curves in compression to calculate the Young's modulus (E, see supplementary material, Sec. 1.2SI and Fig. 8SI) as a largely frequency-independent measure of the material hardness. Similarly to G, although in a more noisy fashion, also the E values scaled well with G′ for all formulations analysed [Fig. 5(a)].

We have also attempted to estimate the Poisson ratio *ν* for these materials, assuming the usual relation *E = *2(1 *+* ν)*G to be valid; the linear fit of E vs. G [Fig. 5(b)] was rather noisy, but it does provide an interesting point: a high Poisson ratio, of about 0.7. This may appear unusual, considering that (incompressible and isotropic) cross-linked networks such as rubber have *ν* = 0.5, but this is not at all surprising for fibrin: its networks, although isotropic, have a negative compressibility;^37^ although fibrin shows syneresis (expulsion of water) at a moderate to high strain, this reduction in volume upon mechanical action can be recorded also at small strains and has been interpreted as stress-induced phase separation, which at a molecular scale corresponds to protein unfolding and aggregation.^38^

In summary, the conclusions of the mechanical analysis are as follows:

1. Bar one sample (1:20 PEG-pep1 at 50 mg/ml) all gels appeared to have a broadly comparable mechanical behaviour (hardening at similar strain, almost completely elastic response, and high Poisson ratio).
2. The very elastic character of the gels and the strong similarity between frequency-independent (creep, G; compression, E) and -dependent (oscillatory, G′) measures further supports the hypothesis that all gels may be significantly cross-linked via covalent bonds.
3. The creep and recovery analysis confirmed that both PEG-pep1 and, to a lesser extent, PEG-pep2 in a dose-dependent fashion reduced the modulus (effect negligible at 12.5, noticeable at 25 and very large at 50 mg/ml fibrin) and introduced a time-dependent, predominantly viscoelastic component to the mechanical behaviour; we interpret this effect as due to the introduction of defects in the self-assembly within and between fibres. However, at this stage, it is not yet possible to ascertain whether PEG-pep1 also influences other molecular regions contributing to the elastic responses of fibrin (supposedly the coiled-coil regions, the γ-chain and the αC domain of fibrinogen^36,39^). Our results align well with the findings of Barker^20,21^ for the a-hole-binding peptide, but not for the b-hole one (in their case increasing the fibrin modulus); there are, however, significant differences between the studies: first, the identity of the b-hole-binding sequence is different [GHRP(GCG) in our case, AHRPYAAC in theirs], and this alters significantly the nature of the interactions; second, we have used a smaller PEG chain (2 vs. 5 kDa), in order to retain the beneficial effect of the macromolecule (minimization of multiple and unspecific binding), but also to reduce its steric hindrance. Indeed, we believe that the stiffening observed by Barker with PEG-AHRPYAAC may have more to do with the PEG size than with the identity of the peptide.
4. PEG-pep1 produced effects of very different magnitude at different fibrinogen concentrations; we are inclined to think that the thicker and less branched fibres of more diluted gels may be more resilient to the defects introduced by the peptide.

In short, both PEG-peptides clearly affected the mechanical performance of the gels, and we are inclined to ascribe this effect to the introduction of defects. In the rest of the study, in view of their use as cell-laden matrices, we have mostly focused only on 6.25 and 12.5 mg/ml gels, because they have the most rapid setting (lowest gel points) and, while preserving an elastic performance, also present features that facilitate cell migration: highest degradability, due to the lower fibrin content, and largest mesh size.

### D. Effect of PEG-peptides on the hydrogel nanostructure

**FIG. 6.**
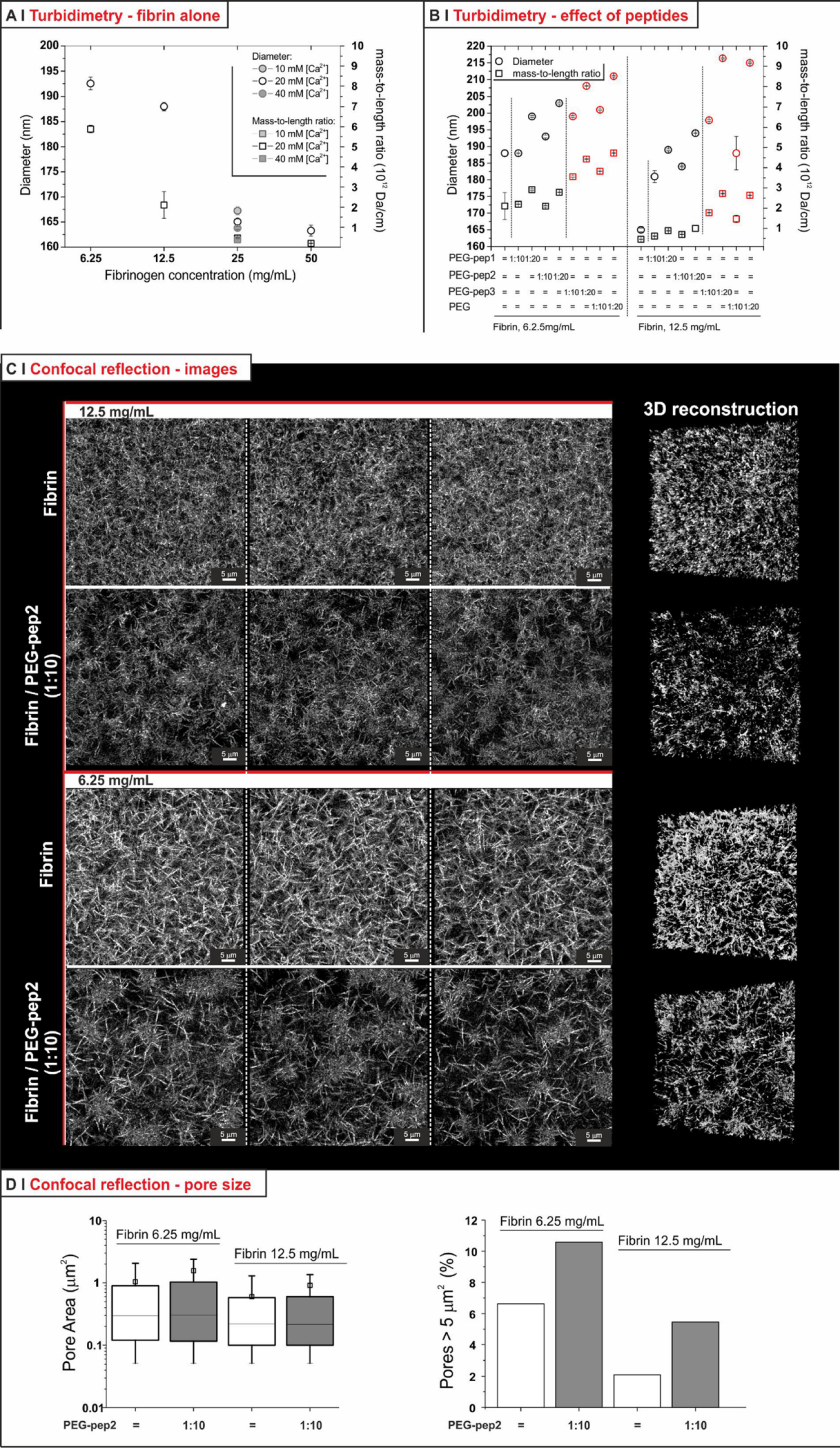
(a) Diameter and mass-to-length ratio of fibres in hydrogels with different fibrinogen (6.25, 12.5, 25, and 50 mg/ml) concentrations produced with 20 mM calcium; the gels at 25 mg/ml were also produce with 10 and 40 mM calcium (*n *=* *3). (b) Diameter and mass-to-length ratio of fibres produced in the presence of different PEG-peptides at a fibrinogen concentration of 12.5 or 25 mg/ml, and with fibrinogen/PEG-peptide molar ratio of 1:10 or 1:20. Results for negative controls (PEG-pep3 and in this case also PEG at the same molar ratio) are shown in red for easy visualization (*n *=* *3). (c) Confocal reflection images of fibrin gels at two different concentrations with and without 1:10 PEG-pep2 (left: confocal sections of different volumes recorded on the same sample for each gel; right: 3D reconstruction). (d) Pore size analysis from confocal reflection images. The pore area [left; the square symbols represent the mean values (±SD), the horizontal lines of the box the 25, 50 (median), and 75 percentile of the distribution] was calculated as described in supplementary material, Sec. 1.2SI (see also Fig. 4SI). The graph on the right hand size shows the percentage of very large (>5 *μ*m^2^) pores.

Turbidity data can be employed to gather information about the characteristics of fibrin fibres, using an approach formulated by Yeromonahos *et al.;*^40^ the interesting feature of this method is that it provides information on fibres in the bulk of an hydrated environment, whereas other techniques such as SEM look at dry materials. We have calculated the hydrogel turbidity τ from optical density measurements [see Sec. 1.2SI and Eq. (5) in supplementary material]; the product τ* λ^5^ has a linear dependency on λ^2^ [see supplementary material, Eq. (6) and Fig. 3SI], and the fibre radius can be obtained from the intercept of such graphs and the mass-to-length ratio from the slope. As previously mentioned, high fibrinogen concentration has been reported to produce thinner and more branched fibres present in higher numbers per volume unit,^31^ and indeed turbidity analysis showed a decrease from >190 nm to around 160 nm in diameter (thinner) and from 6 × 10^12^ to about 1 × 10^12^ Da/cm in mass-to-length ratio (thinner, but also possibly less compact) when increasing the concentration from 6.25 to 50 mg/ml [Fig. 6(a)]; this also fits with our previous observation that less concentrated fibrin gels are more turbid than more concentrated ones^8^ (thicker fibres scatter more than thinner ones).

Incidentally, no significant changes were found in the fibre structure of gels produced in the presence of different calcium concentrations as seen in Fig. 6(a) (which also did not affect the hydrogel modulus, see supplementary material, Fig. 9SI).

In the PEG-peptide-containing samples [Fig. 6(b)], we recorded (1) marginally higher fibre diameters and no effect on the mass-to-length ratio for both PEG-pep1 and PEG-pep2; (2) a slightly higher increase on the fibre diameter and somehow larger mass-to-length ratios when using PEG-pep3 and also non-functional PEG as negative controls. Since the latter can be explained on the basis of partial immiscibility (as for the shorter gel point of PEG-pep3/fibrinogen), it appears that the effect of PEG-pep1 and PEG-pep2 on the fibrin fibres was rather minor.

Confocal reflection showed a higher fibre density with increasing concentration [Fig. 6(c)]: the calculated pore sizes marginally decreased by increasing fibrin concentration, with virtually no effect of the PEG-pep2 on their distribution [Fig. 6(d), left]. The PEG peptide appeared to cause only two effects: an increase in large pores [Fig. 6(d), right] and a reproducible decrease in the scattering intensity [Fig. 6(c)], which may be both due to defects that stop fibre growth or within the fibres: the incorporation of the hydrophilic PEG is likely to decrease the refractive index difference between fibres and the medium, and therefore also the scattering intensity of the former.

**FIG. 7.**
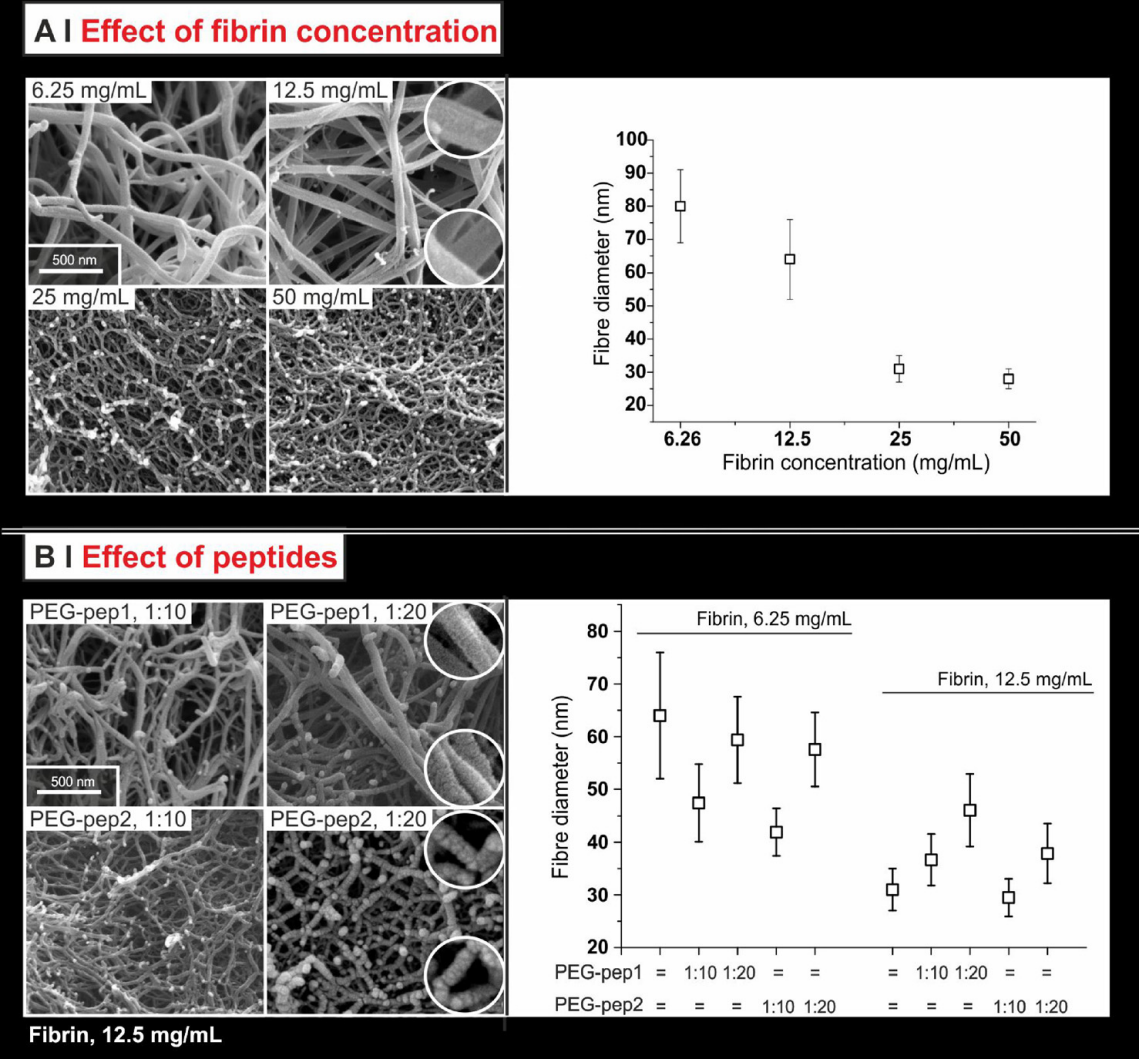
SEM images of fibrin networks (left) and their corresponding fibre diameter measurements (right) for pure fibrin gels [fibrinogen concentrations from 6.25 to 50 mg/ml, (a)] and for modified fibrin gels [12.5 mg/ml fibrinogen concentration + PEG-pep1 or 2 at 1:10 or 1:20 fibrinogen/PEG-peptide molar ratio (b)]. In the circles, magnified particulars are presented to show a different organization/roughness of the fibres produced in the presence of 1:20 PEG-peptides. Note that the diameters obtained from SEM refer to dehydrated fibres, and therefore are necessarily smaller than those recorded through turbidity measurements.

On fibrin, SEM broadly confirmed the two techniques above. The fibre diameter decreased with increasing fibrinogen concentration [Fig. 7(a), right], and the larger number of branched fibres/dead ends [Fig. 7(a), left] paralleled the decrease in mass-to-length ratio.

SEM also showed negligible effects of the PEG peptides on the fibre diameter [Fig. 7(b)], and the difference with turbidity results is to ascribe to defects, which determine an increasing fibre swelling with increasing peptide content. Further, morphological differences can be easily spotted: the fibre surface appeared smooth in the absence of PEG-peptides and seemed to show “stacked rings” (an almost periodic relief) with PEG-pep1 and “blobs” with PEG-pep2. These different features are possibly related to the different kinds of defects that the PEG-peptides may have introduced within individual protofibrils (PEG-pep1) or fibres (PEG-pep2), as a result of their different modes of interaction; at the same time, however, the overall network organization appears to have been left leaving substantially untouched, which confirm the confocal reflection results.

### E. Effect of PEG-peptides on cell migration

**FIG. 8.**
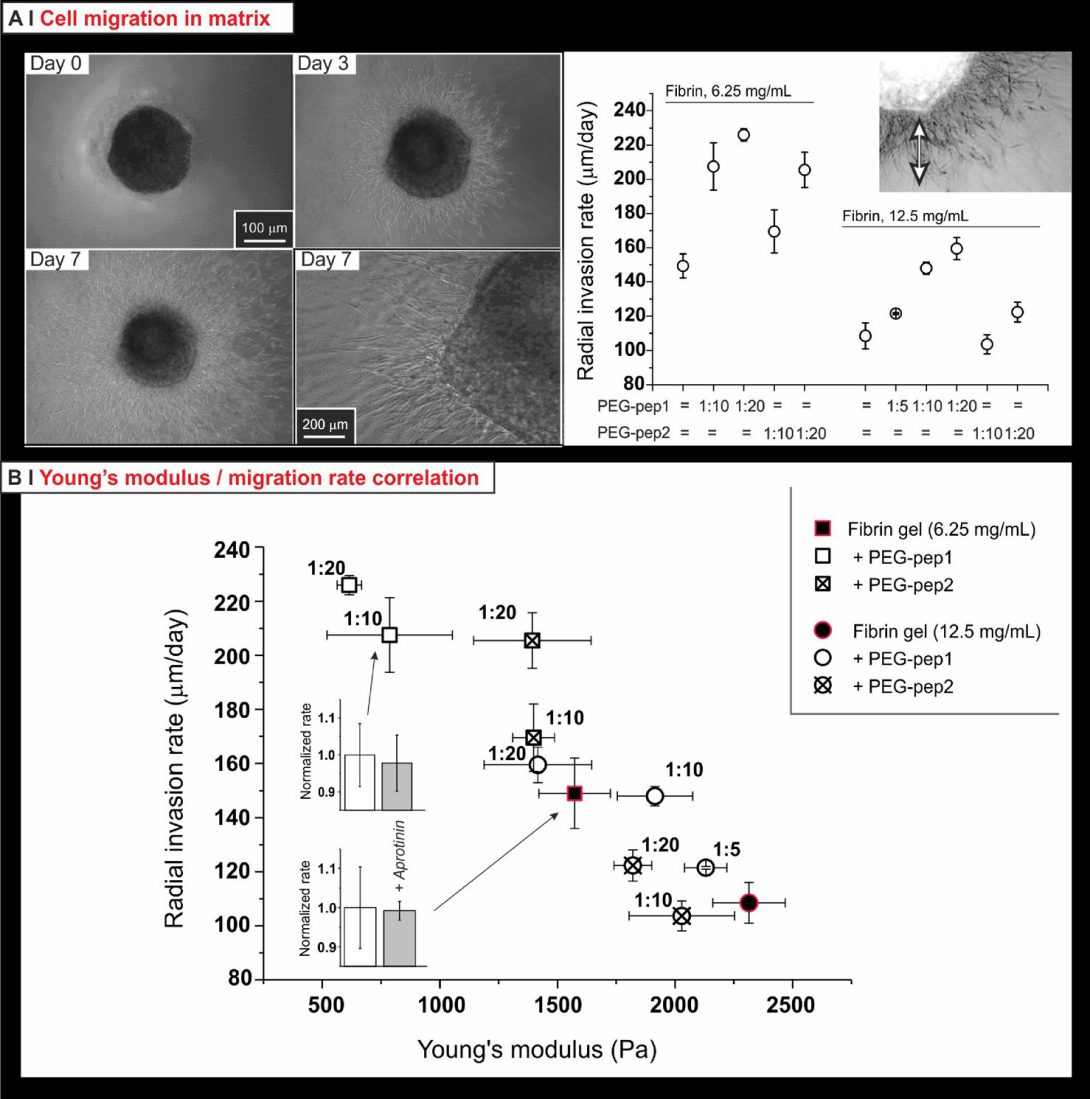
(a) Phase contrast images (left; the bottom right picture provides a high magnification view of the same system in the bottom left picture) and quantification (right) of the radial invasion of HDFa migrating from 1 *μ*l drops of cell-laden fibrin clots (12.5 mg/ml, 30 000 cells/clot) embedded in the fibrin matrices. The rate is calculated from the average distance covered by the invading cell front, divided by time. (b) HDFa migration HDFa in either pure or hybrid fibrin gels well correlated with their softness (the softer, the quicker); importantly, control experiments (220 KIU of aprotinin in the media: grey bars in the two insets on the left) showed the migration not to be based on fibrinolysis.

This migration assay is based on a fibrin-in-fibrin assay: when a volume of HDFa-laden fibrinogen solution is injected into a gelling fibrinogen-based formulation, cells migrate from the clot into the surrounding matrix; the maximum distance covered in the unit time [Fig. 8(a) top and insert in the bottom graph] is a measure of the cell migration capacity. This model is particularly advantageous to assess the migration of cells separately from other phenomena; for example, if seeded homogeneously and/or moving as a coherent front, cells can rapidly remodel and contract the matrix,^41^ and this can profoundly influence the characteristics of their migration too.^42^

First, it is apparent that HDFa migrated more rapidly (almost 2-fold) in less concentrated fibrin gels; second, the presence of the PEG-peptides further facilitated their migration, more for PEG-pep1 than for PEG-pep2, and did so in a dose-dependent fashion; see, for example, the increase in radial invasion rate for 12.5 mg/ml gels with PEG-pep1 in molar ratios of 1:0, 5, 10, and 20 [Fig. 8(a), bottom]. One could have foreseen that the introduction of PEG would have made migration more difficult, because of reduced cell adhesion; on the contrary, whereas formulations with the same PEG content (e.g., 6.25 mg/ml fibrin with 1:20 PEG-peptides) could show different radial invasion rates, increasing the PEG content typically facilitated migration.

From the mechanical and nanostructural analyses presented in Sec. II C, we have concluded that most of these matrices have a similar network organization, but differ in mechanical properties as a consequence of the fibre internal organization/defect density. Therefore, the very strong, linear, but inverse correlation between Young's modulus and radial invasion rate [Fig. 8(b)] suggests that the major determinants of the invasion rate are parameters such as stiffness of individual fibres and inter-fibre adhesion, which would determine the possibility of a cell to “squeeze” through the matrix in a non-proteolytic migration.

This result may seem unexpected: fibroblasts are known to migrate towards/accumulate on stiffer substrates/matrices (durotaxis^43^) while our results may appear to be in contrast to this phenomenon. However, durotaxis is not necessarily related to the speed of locomotion, but to the localization of cells; assuming HDFa to move randomly, if they are slower in matrices higher in modulus, it is there that they will necessarily develop a higher density. Therefore, durotaxis itself may be linked to cell accumulation at sites of lower mobility.

The fact that factor XIII-mediated cross-linking is necessary for a rapid fibroblast migration in fibrin gels^44^ can also be seen as conflicting with our data; however, this motility may depend not on stiffening, but on factors also present in our systems, such as an elastic mechanical response (without cross-linking fibrin is strongly viscoelastic), or on the biochemistry of the cross-links themselves. Further, using aprotinin to reduce fibrin degradation, the same report claimed that fibroblast migration in fibrin gels is hardly based on matrix degradation events (fibrinolysis). Although definitive results may only be reached by studying migration also in the presence of Matrix MetalloProteinase (MMP) inhibitors (aprotinin predominantly inhibits free plasmin and other serine proteases such as trypsin), the virtually identical migration rates that we recorded in the presence and absence of aprotinin [insets in Fig. 8(b)] seemed to confirm the general picture of a mechanically driven process.

## III. CONCLUSION

We have studied the mechanical and nanostructural properties of hybrid PEG/fibrin hydrogels formed via the knob-hole affinity interactions. The type of PEG-peptide, the concentration of fibrinogen and their molar ratios have overlapping roles in influencing the clotting process and consequently the mechanical behaviour. It was shown that the a-hole-binding PEGylated peptide bound fibrinogen more strongly, and slowed gelation, lowered the modulus and introduced a viscoelastic response more easily (= at lower concentrations) than the corresponding b-hole-binding one; however, the latter, although to a smaller extent, produced qualitatively similar results. Since, in general, these effects were not related to major alterations in the elastic nature of the mechanical response, in the strain hardening behaviour, nor in the fibre average dimensions and branching and in the overall network porosity, they were ascribed to structural differences, possibly defects, within the fibres; this would explain the different fibre morphologies seen in SEM. Under this assumption, and considering the predominantly non-fibrinolytic mode of fibroblast migration through fibrin, it can be concluded that a major controlling factor HDFa invasion is the modulus of this matrix (the lower, the faster), most likely the rigidity (bending modulus?) of its fibres.

Clearly this does not completely exclude other effects, e.g., that the incorporation of PEG in the extracellular matrix (ECM) may also affect the binding and presentation of growth factors, and that this on its turn may influence migration. Such effects, however, are difficult to prove, and at the current state of our studies, the correlation with mechanical properties appears to provide the most convincing explanation.

## IV. METHODS

All materials and instruments used for physico-chemical characterization (oscillatory rheology, creep & recovery, compression tests, turbidity, and SEM are reported in the supplementary material, Secs. 1.1SI and 1.2SI).

No ethics approval was required for the experiments described in this study.

### A. Preparation of precursors

#### 1. Synthesis of PEG vinyl sulfone (PEG-VS)

The procedure is reported in supplementary material, Sec. 1.3SI, and the characterization details are provided in Figs. 3SI and 4SI.

#### 2. Synthesis of PEGylated peptides

Solutions of cysteine-containing Pep1, 2, and 3 (corresponding to 38 *μ*mol of thiols for each peptide) were prepared by adding 29.2 mg, 30.8 mg, and 24.3 mg to 9 mL of previously degassed phosphate buffer (150 mM, pH 9.0), respectively. The process was carried out using a 12-position Carousel parallel reactor (Radleys, Saffron Walden, UK) previously purged with argon. In order to reduce the oxidized fraction (potentially present in the commercial cysteine-containing peptides), a modified procedure described by Gailit *et al.*^27^ was used prior to conjugating the peptides onto PEG-VS. Specifically, 1 ml of a 1 M NaBH_4_ solution in 1 M NaOH was added to each peptide solution (corresponding to a final solution concentration of 3.8 mM of peptides and 100 mM of NaBH_4_) and allowed to react for 30 min at 25 °C and 600 rpm. Excess NaBH_4_ was decomposed by acidifying the mixture to pH 3–4 with dropwise addition of a 1 M HCl solution (final volume 3.2 mL) and further stirring for 10 min at 25 °C. The pH was adjusted to a value of 9.0 by adding 1.75 ml of a 1 M NaOH solution, and then the Michael-type addition was started by adding 100 mg of PEG-VS to each peptide solution. Note that the reaction volume was adjusted to 22.2 ml with the addition of phosphate buffer saline (PBS) to obtain a final concentration of 2.15 mM PEG-VS and 1.72 mM of peptides, corresponding to a PEG-VS:thiol molar ratio of 1:0.8. Solutions were stirred at 600 rpm for 3 h under argon at 25 °C, and then 340 *μ*l of mercaptoethanol (ME) was added to each peptide solution and allowed to react for a further 12 h to quench the remaining and unreacted VS groups. The resulting solutions were transferred into dialysis membranes (molecular weight cut-off, MWCO = 2000 g/mol) and dialyzed against milliQ water for 3 days (12 water changes, one every 4 h). The purified products were isolated by lyophilization at standard freeze-drying conditions (−78 °C, 0.05 mbar) for 72 h. ^1^H-NMR spectra, chemical structures, and proton numbering are reported in supplementary material, Fig. 4SI.

##### a. PEG-pep1/ME

Yield = 66 wt. %, white solid, Degree of substitution (DoS) = 100 mol. % (80:20 pep1/ME molar ratio).

^1^H NMR (D_2_O): δ = 4.75–4.63 (m, H9, H15); 4.58–4.51 (m, H19); 4.51–4.43 (t, H11); 4.16–3.95 (m, H3, H3′, H8); 3.95–3.51 (m, H2, H2′, H4, H4′, H5, H5′, H10, H14, H22, H23, HB); 3.43 (s, H1, H1′); 3.33–3.24 (t, H18); 3.25–3.16 (dd, H7a); 3.12–3.03 (m, H6, H6′); 3.03–2.95 (dd, H7b); 2.88–2.81 (t, HA); 2.54–2.24 (m, H12a, H20a); 2.19–1.97 (m, H12b, H20b, H13, H21); 1.97–1.67 (m, H16a, H16b, H17).

MALDI-ToF: positive ion PEG-pep1 ([M]^+^ at *m/z* 2686 ± n × 44.02). See supplementary material, Fig. 5SI.

##### b. PEG-pep2/ME

Yield = 60 wt. %, white solid, DoS = 100 mol. % (86:14 pep2/ME molar ratio).

^1^H NMR (D_2_O): δ = 8.66 (s, H22); 7.35 (s, H21); 4.76–4.63 (m, H9, H15); 4.54–4.43 (t, H11); 4.21–3.96 (m, H3, H3′, H8); 3.96–3.52 (m, H2, H2′, H4, H4′, H5, H5′, H10, H14, H23, HB); 3.43 (s, H1, H1′); 3.27 (s, H18, H20); 3.24–3.15 (dd, H7a); 3.11–3.03 (m, H6, H6′); 3.03–2.94 (dd, H7b); 2.88–2.81 (t, HA); 3.45–2.32 (m, H12a); 2.22–1.97 (m, H12b, H13); 1.97–1.62 (m, H16a, H16b, H17).

MALDI-ToF: positive ion PEG-pep2 ([M]^+^ at *m/z* 2770 ± n × 44.02). See supplementary material, Fig. 5SI.

##### c. PEG-pep3/ME

Yield = 90 wt. %, white solid, DoS = 100 mol. % (70:30 pep3/ME molar ratio).

^1^H NMR (D_2_O): δ = 4.74–4.66 (m, H9); 4.57–4.45 (m, H11, H17); 4.12–3.98 (m, H3, H3′, H8); 3.96–3.90 (m, H16); 3.90–3.53 (m, H2, H2′, H4, H4′, H5, H5′, H10, H14, H20, H21, HB); 3.43 (s, H1, H1′); 3.27–3.14 (dd, H7a); 3.11–3.05 (m, H6, H6′); 3.05–2.97 (dd, H7b); 2.88–2.81 (t, HA); 2.47–2.24 (m, H12a, H18a); 2.2–1.89 ppm (m, H12b, H18b, H13, H19).

MALDI-ToF: Sodiated and protonated PEG-pep3 ([M+Na+H]^+^ at *m/z* 2727 ± n × 44.02 and [M + H]^+^ at m/z 2705 ± n × 44.02, respectively). See supplementary material, Fig. 5SI.

#### 3. Synthesis of fluorescein-labelled (5IAF) peptides

Peptides (1, 2, and 3) were prepared as described earlier at 18 *μ*mol. After reduction with NaBH_4_, a degassed solution of dimethylformamide (DMF) – argon bubbled for 45 min – containing 5IAF (22 *μ*mol, 1.2 eq.s) was added to each 9 ml of peptide solution (1:10 volume ratio), allowing the reaction overnight. The solvent was removed via rotary evaporation and further purified by size-exclusion chromatography using a Sephadex G10 packed column with milliQ water as the mobile phase. Fractions containing pure 5IAF-pep1, 5IAF-pep2, or 5IAF-pep3 were quantified by fluorescence intensity (Synergy 2 multi-mode BIOTek microplate reader) and stored in solution in the dark at 4 °C.

### B. Measurements of binding constants

#### 1. Fluorescence anisotropy (FA) studies

##### a. Saturation experiments

Equal volumes (100 *μ*l) of a labelled peptide solution (5IAF-pep1, 5IAF-pep2, or 5IAF-pep3) in HEPES (4-(2-hydroxyethyl)-1-piperazineethanesulfonic acid)-buffered saline (HBS) and of a fibrinogen solution were mixed together by gentle pipetting into a 96-well plate to obtain final concentrations of fibrinogen ranging from 50 nM to 103 *μ*M, and a constant peptide concentration, i.e., 26 *μ*M for 5IAF-pep1, 25 *μ*M for 5IAF-pep2, or 30 *μ*M for 5IAF-pep3. Note that fibrinogen was previously dissolved in HBS for 2 h at 37 °C at 12 different concentrations. All mixed reagents were incubated for 1 h at room temperature before recording fluorescence polarization values.

##### b. Competition experiments

Fibrinogen/5IAF-pep1 or 5IAF-pep2 complexes were prepared as described in the saturation experiments using 50 *μ*l initial volumes and keeping the fibrinogen concentration fixed. Solutions were pre-incubated for 1 h at room temperature before 50 *μ*l of PEG-peptide 1 or 2 solutions in HBS was added into the wells to obtain a final volume of 150 *μ*l per well. Final concentrations were 36 *μ*M fibrinogen; 0.25 *μ*M 5IAF-pep1 and 0.6 *μ*M to 1.2 mM PEG-pep1; 0.8 *μ*M 5IAF-pep2; and 0.2 *μ*M to 5 mM PEG-pep2. Polarization values were measured after 1 h of incubation at 37 °C. Models used to calculate the binding affinity in both kinds of experiments are reported in supplementary material, Sec. 1.2SI.

#### 2. Isothermal titration calorimetry (ITC) studies

The sample cell for this study can contain up to 270 *μ*l of liquid. Fibrinogen/peptide samples were prepared adding 2 *μ*l aliquots of peptide solution (5.91, 5.92, and 5.73 mM for Pep1, Pep2, and Pep3, respectively) under continuous stirring to 200 *μ*l of 0.175 mM of bovine fibrinogen solution. Fibrinogen/PEG-peptide complexes were prepared adding 2 *μ*l of 1.7 mM of PEG-pep1 or 5.2 mM of PEG-pep2 to 200 *μ*l of 0.012 mM of bovine fibrinogen solution. Note that fibrinogen was prepared as follow: 60 mg of fibrinogen were dissolved in 1 mL HBS for 2 h at 37 °C and then dialyzed (Float-A-Lyzer G2 MWCO: 0.5–1 kDa, Spectrum, Inc.) against HBS for 24 h.

### C. Preparation of fibrin hydrogels

Unless specified, HEPES-buffered saline solution (HBS: 20 mM HEPES; 150 mM NaCl, pH 7.4) was used as a solvent for all preparations; all the precursor solutions were sterile-filtered with a 0.22 *μ*m PES (polyether sulfone) filter (Merck Millipore, Ltd., Cork, Ireland) prior to use. Bovine fibrinogen was dissolved in HBS at 37 °C using an orbital shaker at 200 rpm for 2 h prior use. The fibrinogen and PEG-peptide solutions were mixed and incubated at 37 °C for 30 min under gentle agitation. Thrombin and CaCl_2_ were then gently pipetted together to allow mixing start fibrin polymerization; a library of gels was prepared, keeping constant the final concentration of thrombin (1 U/ml) and CaCl_2_ (20 mM), while varying the final concentrations of fibrinogen (6.25, 12.5, 25.0, or 50.0 mg/ml) and fibrinogen:PEG-peptide molar ratio (1:1, 1:5, 1:10, or 1:20). Fibrin gels containing non-functionalized PEG chains or without PEG were used as controls. Unless specified, all formulations were allowed to gel for 2 h at 37 °C.

### D. Migration assay

Standard cell culture methods are reported in the supplementary material, Sec. 1.4SI. For the migration assays, adult Human Dermal Fibroblasts (HDFa) were gently suspended in 12.5 mg/ml fibrinogen (in HBS) at a final cell density of 3 × 10^7^ cells/ml. 1 *μ*l of cell-loaded suspension was then pipetted within fibrin formulations during gelation in order to constrain the initial spatial location of HDFa. Note that a typical migration assay was prepared using 400 *μ*l of fibrin gel in a 24 multi-well plate. Cells were cultured up to 14 days, changing complete culture media every 5 days. Phase-contrast images of samples were acquired every day using an inverted microscopy (Leica DMI6000) equipped with a 2.5× objective. The cell migration rate was measured by analyzing acquired images with ImageJ (v1.49p, http://rsb.info.nih.gov/ij): the migration area was approximated to a circle and the cell invasion was thus quantified as the variation of the initial diameter, as previously reported by Lutolf *et al.*^28^ An average of nine time points per diameter were measured from day 0 to 8, returning the average migration rate (*μ*m/day). Results (mean and standard deviation) are the average of *n *=* *3 independent experiments.

## SUPPLEMENTARY MATERIAL

See supplementary material for additional information regarding materials and methods; in particular, this includes the description of most physico-chemical characterization methods, including fluorescence anisotropy, isothermal calorimetry, shear rheometry, compression analysis, turbidimetry, confocal reflection microscopy, and scanning electron microscopy (SEM).
